# Using the advantages and avoiding the risks – a public survey about the challenges of online purchases of medicines

**DOI:** 10.1080/02813432.2025.2584902

**Published:** 2025-11-15

**Authors:** A. Persson, M. Troein, U. Jakobsson, S. Lundin, P. Midlöv, C. Lenander

**Affiliations:** ^a^ Department of Clinical Sciences Malmö, Center for Primary Health Care Research, Lund University, Malmö, Sweden; ^b^ Department of Strategic Healthcare Development and Safety, Skåne University Hospital, Malmö, Sweden; ^c^ University Clinic Primary Care, Skåne University Hospital, Region Skåne, Malmö, Sweden; ^d^ Department of Arts and Cultural Sciences, Lund University, Lund, Sweden

**Keywords:** Public health, primary health care, pharmaceutical services, online, professional–patient relations, substandard and falsified medical products, drug-related side-effects and adverse reactions

## Abstract

**Background:**

Around 95% of all websites selling medicines globally are illegal and contribute to the spread of substandard and falsified medicines. Hence, it is important to identify legal pharmacies when accessing medicines online. During 2022–2024, 250 million prescriptions were legally dispensed in Sweden, and 69% were prescribed in primary care. Pharmacists and general practitioners are key healthcare professionals who can guide people to safe online purchases. The overall aim was to describe Swedes’ online purchasing habits for medicines.

**Methods:**

We used a cross-sectional design and collected information from the general population through an annual digital questionnaire for three years in a row, 2022–2024. In total, 6006 respondents, 18–79 years old, were included.

**Results:**

Fifty-two percent had bought medicines online in the last year. Most respondents, 91%, did not recognise the common EU logo mandatory for authorised online pharmacies. People buying over-the-counter medicines online were significantly younger than those buying prescription-only medicines online. Female gender and higher level of education were associated with more online purchases of medicines. Most respondents’ purchasing habits were not influenced by external factors. Reasons for increased online purchases were, for example, that it helps planning regarding access to treatment.

**Conclusions:**

Half of the study participants have used online pharmacies, but fewer than 10% know how to identify a safe online pharmacy, i.e. many are at risk for fraud. Increasing this knowledge by informing those who prefer online purchases has the possibility to use the advantages and avoid the risks associated with online purchases of medicines.

## Introduction

Online pharmacies make it possible to order medicines at any time of day. This accessibility is appreciated by many, and online purchases of medicines are rising [1,2]. The legal framework for online purchases of medicines differs between countries. For example, most countries in the European Union (EU) only permit online purchases of over-the-counter (OTC) medicines, whereas the Nordic countries (Denmark, Finland, Iceland, Norway and Sweden) allow both online purchases of OTC and prescription-only medicines (POMs) [3]. A prerequisite for purchasing POMs legally online is a valid prescription. In Sweden, online pharmacies and e-prescriptions have been available since the beginning of the twenty-first century. This makes Sweden a suitable context for examining the e-commerce of medicines.

Notably, not all websites selling medicines are legal, i.e. authorised by the government. The Alliance for Safe Online Pharmacies (ASOPs) is a non-profit organisation with activities in North America, Europe, Latin America and Asia dedicated to ensuring safe access to medicines and combating illegal online drug sellers. ASOP states that globally, there are around 35,000 active websites selling medicines at any given time, and over 95% are illegal, i.e. unauthorised [4]. Unauthorised online pharmacies contribute to the spread of substandard and falsified (SF) medicines [5]. SF medicines pose a risk to the individual patient, for example, by toxic effect [6] and to society by substandard effect and the risk of contributing to the spread of antimicrobial resistance [7]. Hence, it is important for the public to be able to identify authorised pharmacies to purchase high-quality medicines online.

In the EU, a common logo is mandatory for authorised online pharmacies [8]. Earlier studies have shown that the logo is not common knowledge, neither among the public nor among healthcare professionals [9,10]. This can lead to patients unknowingly putting themselves in danger, thus causing an increased need for healthcare. One way to address the knowledge gap regarding the ability to identify a legal online pharmacy can be through public campaigns, for example, Fight the Fakes or One Pill Can Kill [11,12]. In Sweden, a public campaign on the matter has not been launched since Crime Medicine, 2008 [13]. Another complementary approach can be to educate healthcare professionals, so they can guide people to make safe online purchases of medicines. During 2022–2024, approximately 250 million prescriptions regarding medicines for humans were dispensed by pharmacists in Sweden [14], of which approximately 69% were prescribed in primary health care (personal contact with Henrik Nordin, the National Board of Health and Welfare). General practitioners (GPs) and pharmacists are healthcare professionals who can identify patients who are in situations that increase the likelihood of purchasing medicines online and guide them to safe online purchases. These situations could include the availability of medicines and privacy issues [15,16].

Demographic factors could be another way to identify patients who are more likely to purchase medicines online. A review published in 2024 by Limbu and Huhmann which attempted to compile what influences online purchases of OTC medicines and POMs, concluded that demographic factors among those purchasing medicine online are inconsistent and sometimes contradictory [17]. However, most of the studies included in Limbu’s review (*n* = 31/47) did not specify whether the medicines bought online were OTC medicines or POMs.

The overall aim of this study is to describe Swedes’ online purchasing habits for medicines. To do this we will:

- examine how common online purchases of OTC medicines and POMs are among the general population;
- compare the knowledge of the mandatory EU logo of authorised online pharmacies, as well as compare demographic factors such as age, gender, level of education, and level of income between people buying medicines online with those not buying medicines online;
- explore if, and if so which, situations in the surroundings, i.e. external factors, that appear to influence the buying pattern of medicines online.

## Materials and methods

### Study design

A cross-sectional design was used and information was collected through an annual digital questionnaire that was distributed for three years in a row, 2022–2024. With this repeated design, we were able to see if the results were consistent across all three years.

This study is part of the project ‘Why do we choose the Internet instead of the doctor next door?’ and the manuscript is written in accordance with STROBE guidelines [18,19].

### The SIFO panel

Kantar conducted the survey on our behalf. Kantar is a multinational research and analysis company [20]. Kantar was originally known as ‘the Swedish Institute for Opinion Surveys’ (SIFO; hence the SIFO panel). The SIFO panel is the largest in Sweden with more than 100,000 active members [21]. All panellists are recruited randomly from national population registers. Panellists are contacted to join the panel either by phone call, SMS or mail to ensure reaching as large a part of the population as possible. When being invited, the recruited individual received a registration link via e-mail to set up a panel account. The link is unique and can only be used once to ensure that the invited person, and that person only, can use it for registration. Self-recruitment is not possible.

### The questionnaire

The questionnaire was developed by the research group and reviewed for clarity by Kantar. The questionnaire addressed the following: if online purchases of medicines were made, knowledge of online websites selling medicines, demographic factors (age, sex, level of education, and level of income) and factors influencing the purchasing pattern of medicines (see Appendix I). In 2023 and 2024, the survey was supplemented with the question of whether situations in the surroundings, i.e. external factors, influenced the buying pattern. Kantar digitalised the questionnaire, and the research group tested and approved it via a digital link before it was distributed.

### Data collection

The questionnaire was sent out using a Computer Assisted Web Interview (CAWI) methodology. The survey was carried out for a couple of weeks in the spring each year. A minimum of 2000 randomly selected persons from the SIFO panel answered the digital questionnaire, and each year a new sample was drawn; hence, it is not the exact same respondents in the three surveys. All respondents were Swedish residents aged 18–79 years old. The respondents were stratified by gender, age and residential area before being invited to the questionnaire, as well as after answering the questionnaire, to mirror the Swedish population. All information was self-reported except residential area, which was based on current postal code. Since the classification of medicines (OTC medicines and POMs) was made by the respondents, we tried to minimise errors by including the option of dietary supplements to decrease the risk for respondents to mix dietary supplements with OTC medicines.

### Analysis

The results were analysed using SPSS, version 28.0.1.1 (SPSS Inc., Chicago, IL). Respondents who reported buying medicines online were divided into three groups: only buying OTC medicines, buying both OTC medicines and POMs, and only buying POMs.

Comparisons of the continuous variable age were analysed with analysis of variance, ANOVA, while categorical variables such as gender were analysed using Chi-square test. An alpha level of 5% was considered significant.

Answers to the open-ended question regarding external factors, which had influenced the buying pattern of medicines online, were grouped according to content. The different groups were tagged with labels.

Since the analyses from the different years showed a similar pattern, we chose to present the results from all three surveys combined to increase the power of the study.

Respondents who reported only buying dietary supplements online were excluded from the analyses, where comparisons between online buyers and non-buyers were made, since dietary supplements are not authorised medicines.

### Definitions of variables

#### Online buyers

Online buyers in this study are defined as people who reported buying medicines online.

#### Non-buyers

Non-buyers in this study are defined as people who reported not buying medicines online. These people might or might not buy medicines from community pharmacies.

#### Gender

The respondents answered the question ‘What is your legal gender?’ and the answer options were male, female or do not want to disclose.

#### Level of education

Low education was considered to be present if the highest completed level of education was primary school. High education was considered to be present if the highest completed level of education was a post-secondary education. Everyone in between was defined as middle education.

#### Level of income

Low income was defined as an income of less than 1900 EUR monthly. This is in accordance with Eurostat’s ‘At risk of poverty’, which uses 60% of the countries’ median income, and OECD which uses 50% of the countries’ median income as threshold [22,23]. High income was defined as an income of 5000 EUR monthly or higher. This is in accordance with the Swedish threshold, when the tax level increases from approximately 30 to 50%. Everyone in between was defined as middle income.

According to Statistics Sweden, 45% of the Swedish population have a post-secondary education, while primary school is the highest completed level of education for 10% of the Swedish population [24]. Approximately, 30% of the Swedish population over 20 years of age have a monthly income below 1900 EUR, and 10% have a monthly income above 5000 EUR.

### Ethics

The survey was approved by the Swedish Ethical Review Authority in October 2021 (reference number 2021-05062) and was conducted in 2022. An amendment was approved in March 2023 to conduct the surveys in 2023 and 2024 (reference number 2023-01671-02). The surveys were performed in accordance with the principles stated in the Declaration of Helsinki. All respondents gave their consent to participate, and they were informed that they could always choose to refrain from answering any questions or stop participating if they wanted. The respondents were rewarded with points for participating. The points could be redeemed for cinema tickets, magazines, etc., but not for cash.

Surveys by Kantar are conducted in line with the EU General Data Protection Regulation (GDPR). Data are stored within Kantar for up to two years and only within the EU/EEA [25]. A pseudonymised dataset was delivered by Kantar to the research group, and all analyses were made by the researchers. The respondents’ identities and postal codes were kept unknown to the researchers. The dataset will be securely stored for 15 years at Lund University.

## Results

In total, 6006 respondents were included with an overall response rate of 26%. Details from each year are shown in Table 1. Since respondents were stratified by gender and age, 50% were men, and the average age was 49 years (range 18–79 years). Two-thirds had completed post-secondary education, and an equal amount, i.e. two-thirds, had a monthly income regarded as a middle income in Sweden; for more detailed information (see Table 2). A total of 62% of the respondents were working. Among those who did not work, 24% were retired, 8% studied, and the rest were either unemployed (2%), on sick leave (1%) or on parental leave/other (3%).

### Description of how common online purchases of OTC medicines and POMs are among the general population

In total, 52% of the respondents reported that they had bought medicines online in the last 12 months (*n* = 3099). More specifically, just over 28% of the respondents only bought OTC medicines (*n* = 1688), just over 7% only bought POMs (*n* = 440), and the remaining 16% bought both OTC medicines and POMs (*n* = 971). Around one-third, 37%, reported that they had not purchased any medicines online in the last 12 months (*n* = 2223), and the remaining 11% reported purchasing only dietary supplements (*n* = 684).

### Comparison of buyers and non-buyers of medicines online regarding knowledge of the mandatory EU logo of authorised online pharmacies

The vast majority of all respondents, 91%, reported that they had not seen the mandatory EU logo for authorised online pharmacies before (*n* = 5470). The knowledge of the logo was significantly higher among those reporting buying medicines online (*n* = 420) compared to those reporting not buying medicines online (*n* = 68), 14% versus 3%, *p* < 0.001. Of all respondents, around 90% stated that it was quite or very important for them that the medicines they bought were approved by Swedish authorities (*n* = 5477). Despite this, 51% of those who bought medicines online did no such control before their purchase (*n* = 1592).

### Comparison of buyers and non-buyers of medicines online regarding demographic factors such as age, gender, level of education and level of income

**Figure 1.**
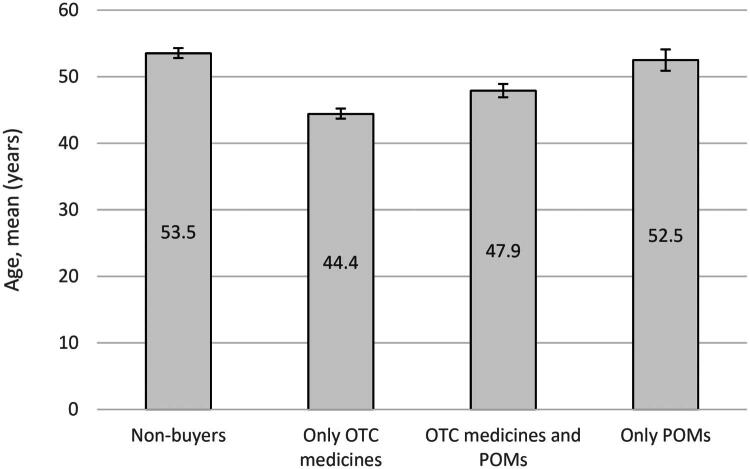
Description and comparison with ANOVA between non-buyers and the three groups of buyers of medicine online regarding age expressed as mean age with 95% confidence interval.

Figure 1 shows that non-buyers have a significantly higher mean age than buyers buying OTC medicines with or without POMs, but the mean age does not differ significantly between non-buyers and respondents only buying POMs. All three subgroups of buyers, i.e. only OTC medicines, OTC medicines and POMs, and only POMs, significantly differ from each other.

Women bought medicines online to a significantly higher degree than men, as shown in Table 3. Respondents with high or middle level of education bought medicines to a significantly higher degree than respondents with low education. Level of income did not significantly differ between buyers and non-buyers.

### External factors influence on the buying pattern

In total, 4002 respondents answered this question since it was not available in the 2022 survey. Most respondents, 61%, stated that situations in the surroundings, i.e. external factors, did not influence their buying pattern (*n* = 2440), 9% stated that external factors had led to increased online purchases (*n* = 373), and 1% stated that external factors had led to decreased online purchases (*n* = 49). Of the remaining respondents, 23% never bought medicines online (*n* = 918), and the rest were hesitant. In an additional open-ended question, the respondents with affected purchasing habits (*n* = 422) could elaborate on what external factors influenced them; a total of 236 respondents took this opportunity. The external factors mentioned in the open-ended question as reasons for increased online purchases of medicines were grouped according to content. The different groups were tagged with labels. The four categories, presented in italic, are exemplified with a quote. The quotes have been translated by the authors.

The *risk of getting an infection* in local pharmacies (with the covid-19 pandemic as the main external factor) was one of the categories.

I would say that the covid-19 pandemic changed the way I shop, partly because of the risk of getting an infection in local pharmacies, but also due to the improved system with faster deliveries. (Male 30–49 years)

Another category was *financial factors*, where lower prices online, mainly OTC medicines, were mentioned together with increased inflation and high fuel prices.

Expensive fuel prices. The nearest pharmacy is 20 km away. Also, it is more expensive to buy medicines in a local pharmacy than online. Shipping fee is included. (Female 65–79 years)

Respondents also described that online pharmacies could be a way to have *better forward planning* when it comes to accessing their treatment (because of global conflicts, increased risk of medicine shortages and the respondents’ perception that local pharmacies have limited stock compared to online pharmacies).

The covid-19 pandemic and disorder in the surrounding area demand a larger supply of medicines at home (preparedness). (Male 30–49 years)

Also, the respondents expressed that online purchase of medicines *makes life easier* (takes less time and is available around the clock).

I’ve become more ill, so it is hard to get out. (Female 50–64 years)

The external factors mentioned in the open-ended question as reasons for decreased online purchases of medicines were also grouped according to content and tagged with labels. Two categories for decreased online purchases emerged: the *desire to support local pharmacies* together with *safety aspects* (as the fear of getting SF medicines or falling victim to theft of personal information).

I want to support local businesses so that they remain in these grim times. (Female 65–79 years)

I have become more aware of fraud and fake medicines online. (Male 50–64 years)

## Discussion

This study showed that buying medicines online was a common occurrence among Swedes. Despite this, the vast majority did not recognise the mandatory EU logo used by authorised online pharmacies. This knowledge gap increases the risk that medicines are bought from unauthorised pharmacies and SF medicines might be delivered. This study also shows a significant difference between online buyers and non-buyers regarding the demographic factors of age, gender, and level of education, while no significant difference could be seen regarding level of income. The majority did report that their buying pattern was unaffected by external reasons.

In this study, every other respondent had purchased medicines online in the last 12 months. Studies from other European countries show prevalence of the same magnitude, for example, 55% in Poland, Hungary, Czech Republic and Slovakia [2]. In these countries, only OTC medicines are legal to buy online. Purchases of OTC medicines were more common than POMs in our study. The sample in this study mirrors the general population in Sweden, not patients with a chronic condition who use medicines regularly. In comparison with a report from ASOP EU [26], where 63% of the respondents had a chronic condition and 30% were caregivers of a person with a chronic condition, 56% of the respondents from Sweden stated that they had bought POMs online, compared to 23% in this study. This indicates a higher share of online purchases of POMs among people with a chronic condition compared to the general population.

We have previously shown that the mandatory EU logo for authorised online pharmacies is not well-known either among the public or by professionals working with medicines [9,10]. This study confirms our previous findings. In agreement with ASOP’s report, we have shown that the logo is recognised more frequently by online buyers than non-buyers, but there is room for improvement to ensure safe purchases of medicines online. In Sweden, the government has commissioned the Medical Product Agency to carry out a public campaign to increase knowledge of safe online medicine purchases [27].

Demographic factors among those purchasing medicine online have been reported as inconsistent and sometimes contradictory [17]. A possible explanation could be that different samples are used in the included articles in the review. Our approach with 2000 respondents stratified by gender and age, three years in a row, showing the same results, confirms that:

- The average age for people buying OTC medicines online was significantly lower than that of those buying POMs online. Non-buyers had the highest average age.
- Female gender was associated with more online purchases of medicines, especially OTC medicines.
- A higher level of education was associated with more online purchases of medicines, especially OTC medicines.
- Level of income did not seem to be significantly associated with online purchases of medicines.

Regarding age and gender, our results are in line with a recently published study from Denmark, which showed that females and patients aged 30–69 years old more often chose online pharmacies, while men and patients over 70 years of age more often chose community pharmacies [28].

Even though we were able to show significant differences regarding age, gender and level of education, the differences are small, and the results are hard to apply in a real-world setting, for example, when a person is meeting a GP or a pharmacist. However, the results may be valuable when planning a public campaign to address the main target group.

Online purchases of medicines were common among the general population and probably even more common among people with a chronic condition, and therefore have regular use of medicines. It is relevant to ask people – especially those with continuous use of medicines – if they buy medicines online and then guide them to make safe online purchases.

As a complement to demographic factors associated with more online purchases, some respondents expressed external factors which influenced their purchasing habits. A common external factor mentioned by the respondents, which increased access to medicines from online pharmacies, was the covid-19 pandemic. The restrictions during the pandemic created a new habit among people, which many have kept for convenience, lower cost, and to avoid the risk of getting an infection when visiting a community pharmacy. Fittler et al. conducted a cross-sectional study in Poland, Czech Republic, Hungary and Slovakia, before and after the covid-19 pandemic, examining the prevalence of online purchases of OTC medicines, which also showed an increase in online purchases of medicines after the pandemic compared to before [2].

The respondents also mentioned that online purchases can contribute to better forward planning. Some mentioned that they experienced that community pharmacies have a limited stock compared to online pharmacies, leading to two visits, which takes time and costs money. The respondents also mentioned a concern due to war in the surrounding area, which increased the urge to keep medicines in stock at home. Shortages of medicines can lead to frustration for both patients and health professionals and is a time thief. In our previous study [16], we highlighted shortages as one factor which may lead to online purchases. Moreover, limited access to medicines is a contributing factor to the spread of SF medicines, according to WHO [29]. All this taken together is in line with the study by Nymberg et al. where patients saw clear advantages with e-health as a complement but not a substitute to regular care [30]. The expressed will to increase forward planning may increase compliance with prescribed treatment as well as save time for both patients and healthcare. We believe that healthcare should encourage people to take action as regard accessing their medicines, and this could be one situation where healthcare can inform about safe online pharmacies to refill prescriptions.

Overall, the majority stated that external factors did not influence their buying patterns. Some prefer online pharmacies, others do not. As expressed by the respondents in this study, if you have a chronic condition that makes it hard to visit a community pharmacy or if you live in a rural area, online pharmacies might provide advantages. On the other hand, if you live near a community pharmacy and/or if you feel insecure about online purchases, community pharmacies are perhaps a better option.

### Strengths and limitations

Cross-sectional studies show a snapshot of reality and cannot show causation, only association. Cross-sectional studies are a good way to answer questions about prevalence and describe features of a population [31]. A limitation of this study was that the data were self-reported, and the distinction between OTC medicines and POMs was made by the respondents. We tried to help respondents by including dietary supplements as an option as well as including the word health-related products to signal that all preparations for health are not approved as medicines. In accordance with Kantar, we consider the response rate acceptable [32]. As always, there is a risk of selection and information bias when a survey is conducted. The oldest respondent in this survey was 79 years old due to predefined selection, and it would be valuable to include even older respondents in future studies. Also, conducting a digital survey about online purchases will select digital respondents, possibly overestimating the prevalence of online purchases. In Sweden, 95% of the population over the age of 16 uses the Internet [33]. The SIFO panel mirrors the Swedish population since the panellists were randomly recruited from national registers. We also had a large sample, *n* > 2000, from three different years, which showed the same pattern and validates the result. The large sample also reduces the risk that misclassification of OTC medicines and POMs will affect the result.

In future studies, it would be interesting to gather information about health status among respondents, including if they use POMs regularly. It would also be interesting to ask if they bought POMs with or without a valid prescription online. Another area of interest would be to compare persons living in rural areas with those living in urban areas. Co-researchers working in our project ‘Why do we choose the Internet instead of the doctor next door?’ have conducted a qualitative study to examine the reasons that may lie behind online medication purchases [34].

## Conclusions

Online pharmacies can be a valuable complement to community pharmacies and are already widely used in Sweden. Since this study showed the common nature of accessing medicines online among the public, we believe that demographic factors could be a way of finding target populations for public campaigns rather than in personal encounters. We believe that a personal encounter with a GP or a pharmacist offers an opportunity to enquire about the patient’s preferences concerning how to buy medicines. If the patient wants to use online pharmacies, the health professional has the opportunity to give information about safe procedures. Extra attention needs to be given to people in risk situations, for example, with prescribed medicines that risk being out of stock.

We suggest that online pharmacies can be a way to increase forward planning and secure access to the prescribed treatment. This should be encouraged since it may increase compliance and save resources in healthcare. For safety reasons, it is important to guide people to safe online purchases of medicines to avoid fraud, i.e. SF medicines or identity theft, for example, by increasing the knowledge about the mandatory EU logo used by authorised pharmacies.

If we use the advantages and avoid the risks with online purchases of medicines, resources in healthcare can be saved.

## Supplementary Material

Main document_Clean copy Final.docx

## Data Availability

The data underlying this article will be shared upon reasonable request to the corresponding author.

## Appendix I

### Questionnaire Kantar Public

#### Target group: The Public

**Table:**

1. *During the last 12 months, did you search for medicines or health-related products online for yourself or for a relative?*
Yes
No
Do not know
2. *Where do you find information online about medicines?*
Select all options that apply:

Online pharmacies
FASS (Swedish version of Physician’s Desk Reference)
Social media (for example, Facebook)
YouTube
Google
Forum (for example Flashback or Familjeliv)
Sites recommended by physicians or other healthcare staff
Sites recommended by pharmacy staff
Sites recommended by friends or acquaintances
Other:_____
Do not know
*Filter: Selected online pharmacies above*
3. *On which online pharmacy/pharmacies did you find information about medicines or health-related products?*
Provide your answer:___________
Do not know/Do not want to state

4. *During the last 12 months, did you buy medicines or health-related products online for yourself?*
Select all options that apply:

Yes, OTC medicines
Yes, prescription-only medicines, for example, antibiotics and painkillers
Yes, dietary supplements, for example, vitamins
No
Do not know

*Filter: Did buy something online*
5. *Please state what you bought?*
Provide your answer:_________
Do not know/Do not want to state
*Filter: Did buy something online*
6. *How do you find websites where you buy medicines or health-related products?*
Select all options that apply:

Google
Social media, for example, Facebook
Flashback, familjeliv or similar sites
Sites recommended by physicians or pharmacy staff
Sites recommended by friends or acquaintances
Other:_____
Do not know
*Filter: Did buy something online*
7. *In what language was the website where you bought medicines or health-related products?*
Select all options that apply:

Swedish
English
Other language:_______
Do not want to state
Do not know
*Filter: Did buy something online*
8. *At your last purchase online of medicines or health-related products, did you in any way check if the site was authorised by government?* Select all options that apply:

Yes, through the common EU logo for authorised pharmacies
Yes, through______________
No, I did not control
Do not want to state
*Filter: Did buy something online*
9. *Please comment on your perceived effect of the medicines or health-related products you bought online. Did it work as intended? Did you experience any problems? Other comments?*
Provide your answer:___________
Do not know/Do not want to state

Filter: Did buy something online
10. *Do you know if the medicines or health-related products you bought were approved by the Swedish government and were legal to sell in Sweden?*
Yes, I know it was approved and legal to sell
Yes, I think it was approved and legal to sell
No, I do not think it was approved and legal to sell
No, I know it was not approved and legal to sell
Do not know

11. *Have situations in your surroundings in any way affected your purchasing patterns of medicines or health-related products in the last 12 months? (For example, the covid-19 pandemic, the war in Ukraine, shortage of your medicine or health-related product, etc.)*
Yes, I purchase more medicines or health-related products online today
Yes, I consider purchasing medicines or health-related products online in the future
Yes, I purchase fewer medicines or health-related products online today
Yes, I consider purchasing fewer medicines or health-related products online in the future
No, unaffected
No, I never purchase medicines or health-related products online
Uncertain, do not know

*Filter: Affected purchasing pattern*
12. *Which situation in your surroundings has, during the last 12 months, affected your purchasing patterns?*
Provide your answer:
Do not know/Do not want to state
13. *Overall, does it matter to you that medicines or health products you buy online are approved by the Swedish government?*
Yes, it is very important
Yes, it is fairly important
No, it is not very important
No, it is not important at all
Uncertain, do not know

14. *Have you seen this symbol/logo before?*
Yes
No

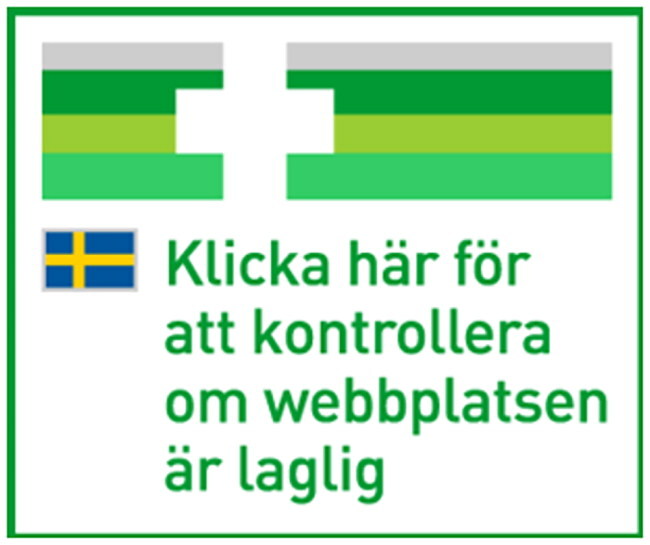

15. *How did you learn to search and review information online (source criticism)?*
Self-taught
Taken courses as an adult
Learned in school
Have not learned to search and review information online (source criticism)
Do not know

### Background questions

**Table:**

*Gender* Male/Female/Do not want to disclose
*Age ___*
*What is your occupation?*
Worker
Civil servant
Self-employed
Retired
Student
Unemployed
On sick leave
On parental leave
Other
Do not know

*Which is your highest education?*
Have not completed primary school
Primary school
2-year secondary school
3-year secondary school
Post-secondary education, not university
University, two years or less
University, more than two years
Do not know/Do not want to state

*How much is your personal monthly income?* With income, we mean before tax. Student aid, housing supplement, pension, etc., also count as income.

Have no income
Less than 800 EUR/month
800–1399 EUR/month
1400–1899 EUR/month
1900–2499 EUR/month
2500–2999 EUR/month
3000–3499 EUR/month
3500–4199 EUR/month
4200–4999 EUR/month
5000–6499 EUR/month
6500–8399 EUR/month
8400–9999 EUR/month
10,000 EUR/month or more
Do not know/Do not want to state

*Which political party would you vote for if a general election took place today?*

Centre Party (Centerpartiet)
Christian Democrats (Kristdemokraterna)
Feminist Initiative (Feministiskt initiativ)
Green Party (Miljöpartiet)
Left Party (Vänsterpartiet)
Liberals (Liberalerna)
Moderate Party (Moderaterna)
Sweden Democrats (Sverigedemokraterna)
Swedish Social Democratic Party (Socialdemokraterna)
Other: *OPEN
I vote blank
I would not vote
I am not allowed to vote
Do not want to state
Do not know

**Table 1.** Description of the inclusion process of respondents.

	2022	2023	2024	Total
Invited to participate, *n*	8519	10,060	7483	26,062
Opened survey, *n*	2263	2327	2189	6779
Response rate, %	27	23	29	26
Opted out, *n*	121	151	185	457
Excluded due to full quota, *n*	138	176	2	316
Included, *n*	2004	2000	2002	6006

**Table 2.** Baseline characteristics of all respondents.

		2022	2023	2024	Total
Respondents (*n*)		2004	2000	2002	6006
Gender	Male, *n* (%)	1002 (50)	1001 (50)	1018 (51)	3021 (50)
Age (years)	Mean (SD)	48 (17)	48 (17)	52 (18)	49 (17)
	Median (IQR)	48 (28)	48 (29)	55 (31)	49 (30)
Highest completed education, *n* (%)	Primary education or less	78 (4)	90 (5)	98 (5)	266 (4)
	Secondary education	586 (29)	592 (30)	577 (29)	1755 (29)
	Post-secondary education	1322 (66)	1304 (65)	1316 (66)	3942 (66)
	Don’t know/don’t want to disclose	6 (<1)	14 (<1)	11 (<1)	31 (<1)
	Missing	12 (<1)	0 (0)	0 (0)	12 (<1)
Monthly income, *n* (%)	<1900 EUR	374 (19)	391 (20)	335 (17)	1100 (18)
	1900–5000 EUR	1224 (61)	1195 (60)	1208 (60)	3627 (60)
	>5000 EUR	226 (11)	262 (13)	304 (15)	792 (13)
	Don’t know/don’t want to disclose	168 (8)	152 (8)	155 (8)	475 (8)
	Missing	12 (<1)	0 (0)	0 (0)	12 (<1)

**Table 3.** Description and comparison with Chi-square test between non-buyers and the three groups of buyers of medicine online regarding gender, level of education, and level of income expressed as percentages.

			Buyers			
		Non-buyers	Only OTC medicines	OTC medicines and POMs	Only POMs	*p* Value
Gender, *n* (%)	Female	910 (35)	906 (34)	598 (23)	221 (8)	<0.001
	Male	1313 (49)	782 (29)	373 (14)	219 (8)	
Level of education, *n* (%)	High education	1411 (40)	1138 (32)	675 (19)	279 (8)	<0.001
	Middle education	659 (42)	499 (32)	260 (17)	136 (9)	
	Low education	136 (58)	41 (18)	32 (14)	24 (10)	
Level of income, *n* (%)	High income	311 (44)	210 (30)	142 (20)	45 (6)	0.163
	Middle income	1303 (40)	1065 (33)	585 (18)	268 (8)	
	Low income	408 (42)	297 (31)	175 (18)	84 (9)	
